# Cellular Uptake, DNA Binding and Apoptosis Induction of Cytotoxic *Trans*-[PtCl_2_(*N,N*-dimethylamine)(Isopropylamine)] in A2780cisR Ovarian Tumor Cells

**DOI:** 10.1155/MBD.2001.29

**Published:** 2001

**Authors:** José M. Pérez, Eva I. Montero, Adoración G. Quiroga, Miguel A Fuertes, Carlos Alonso, Carmen Navarro-Ranninger

**Affiliations:** 1 Departamento de Química Inorgánica Facultad de Ciencias Universidad Autónoma de Madrid, Cantoblanco Madrid 28049 Spain; 2 Centro de Biología Molecular “Severo Ochoa”(CSIC-UAM) Facultad de Ciencas Universidad Autónoma de Madrid, Cantoblanco Madrid 28049 Spain

## Abstract

*Trans*-[PtCl_2_(N,N-dimethylamine)(isopropylamine)] is a novel *trans*-platinum compound that shows cytotoxic activity in several cisplatin resistant cell lines. The aim of this paper was to analyse, by means of molecular cell biology techniques and total reflection X-ray fluorescence (TXRF), the cytotoxic activity, the induction of apoptosis, the cellular uptake and the DNA binding of *trans*-[PtCl_2_(N,N-dimethylamine)(isopropylamine)] in the cisplatin resistant cell line A2780cisR. The results show that this drug is more cytotoxic and induces a higher amount of apoptotic cells than cisplatin in A2780cisR cells. However, the intracellular accumulation and extent of binding to DNA of *trans*-[PtCl_2_(N,N-dimethylamine)( isopropylamine)] is lower than that of *cis*-DDP. Moreover, *trans*-[PtCl_2_(N,N-dimethylamine)(isopropylaminae)] is partially inactivated by intracellular levels of glulathione. The result suggest that circumvention of ciplatin resistance by *trans*-[PtCl_2_(N,N-dimethylamine)(isopropylamine)] in A2780cisR cells might be related with the ability of this drug to induce apoptosis.


### CELLULAR UPTAKE, DNA BINDING AND APOPTOSIS INDUCTION OF CYTOTOXIC *TRANS*-[PtCl$_2$(*N,N*-DIMETHYLAMINE)(ISOPROPYLAMINE)] IN A2780cisR OVARIAN TUMOR CELLS

José M. Pérez[1], Eva I. Montero[1], Adoración G. Quiroga[1], Miguel A Fuertes[2], Carlos Alonso[2] and Carmen Navarro-Ranninger*[1]

[1] Departamento de Química Inorgánica, Facultad de Ciencias, Universidad Autónoma de Madrid, Cantoblanco, 28049-Madrid, Spain carmen.navarro@uam.es.

[2] Centro de Biología Molecular "Severo Ochoa"(CSIC-UAM), Facultad de Ciencias, Universidad Autónoma de Madrid, Cantoblanco, 28049-Madrid, Spain.

### ABSTRACT

*Trans*-[PtCl$_2$(*N,N*-dimethylamine)(isopropylamine)] is a novel *trans*-platinum compound that shows cytotoxic activity in several cisplatin resistant cell lines. The aim of this paper was to analyse, by means of molecular cell biology techniques and total reflection X-ray fluorescence (TXRF), the cytotoxic activity, the induction of apoptosis, the cellular uptake and the DNA binding of *trans*-[PtCl$_2$(*N,N*-dimethylamine)(isopropylamine)] in the cisplatin resistant cell line A2780cisR. The results show that this drug is more cytotoxic and induces a higher amount of apoptotic cells than cisplatin in A2780cisR cells. However, the intracellular accumulation and extent of binding to DNA of *trans*-[PtCl$_2$(*N,N*-dimethylamine)(isopropylamine)] is lower than that of *cis*-DDP. Moreover, *trans*-[PtCl$_2$(*N,N*-dimethylamine)(isopropylamine)] is partially inactivated by intracellular levels of glutathione. The results suggest that circumvention of ciplatin resistance by *trans*-[PtCl$_2$(*N,N*-dimethylamine)(isopropylamine)] in A2780cisR cells might be related with the ability of this drug to induce apoptosis.

### INTRODUCTION

Cisplatin [*cis*-diamminedichloroplatinum (II), *cis*-DDP] is one of the most widely used drugs in the treatment of cancer. It shows remarkable activity alone or in combination with other drugs in the treatment of several tumors, including those of the lung, ovary, testes and bladder [1, 2]. The success of *cis*-DDP as an antitumor drug has been attributed to different factors, including penetration of the cellular membrane, accumulation in the tumor cell, and efficiency in coordinating with chromosomal DNA [3, 4]. The extent of DNA lesions induced by *cis*-DDP in the cell nuclei and the cell killing potential of the drug is believed to depend on the cellular level of reactive platinum species and the persistence of platinum within the cells [5, 6]. Despite the success of *cis*-DDP against certain types of cancer, the patients treated with the drug suffer from severe side effects including nephrotoxicity, nausea and vomiting, myelosuppression and ototoxicity [2, 3]. Moreover, quite often tumors become resistant to cisplatin [7]. The *cis*-DDP resistance may take place at various levels, including cellular accumulation, interaction with glutathione (GSH) and/or metallothioneins, DNA repair and defective apoptotic program [8].

Although transplatin or *trans*-DDP (the stereoisomer of cisplatin) is clinically inactive, several classes of *trans*-platinum complexes show antitumor activity and some of them are able to circumvent cisplatin resistance [9]. *Trans*-[PtCl$_2$(*N,N*-dimethylamine)(isopropylamine)] (Figure 1) is a novel *trans*-platinum(II) complex with mixed aliphatic amine ligands which show cytotoxic activity in tumor cell lines sensitive and resistant to cisplatin [10]. Moreover, *trans*-[PtCl$_2$(*N,N*-dimethylamine)(isopropylamine)] induces tumor cell death through apoptosis [11]. The A2780cisR human ovarian tumor cell line may be considered a model to study cisplatin-resistance because it exhibits acquired resistance to *cis*-DDP from a combination of decreased uptake, enhanced DNA repair/tolerance and elevated GSH levels [12, 13]. The results reported in this paper indicate that *trans*-[PtCl$_2$(*N,N*-dimethylamine)(isopropylamine)] is able to overcome cisplatin-resistance in A2780cisR cells through apoptosis induction. *Trans*-[PtCl$_2$(*N,N*-dimethylamine)(isopropylamine)] induces a higher percentage of apoptotic cells than *cis*-DDP both in A2780 and A2780cisR cell lines. However, the intracellular accumulation and extent of binding to DNA of *trans*-[PtCl$_2$(*N,N*-dimethylamine)(isopropylamine)] in A2780cisR cells is lower than that of *cis*-DDP. Interestingly, the cytotoxic activity and the binding to DNA of *trans*-[PtCl$_2$(*N,N*-dimethylamine)(isopropylamine)] in A2780cisR cells increases by previous cell treatment with the GSH inactivator, L-buthionine sulfoximine (L-BSO) suggesting that the drug is partially inactivated by GSH.

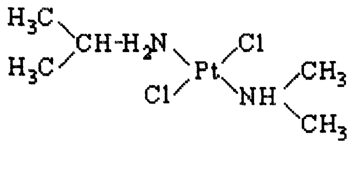

*Trans*-[PtCl$_2$(*N,N*-dimethylamine)(isopropylamine)]
M.W.=370.18

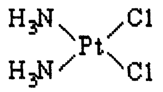

CISPLATIN
M.W.= 300

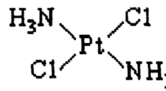

TRANSPLATIN
M.W.= 300

**Figure 1.** Structures and molecular weights of *trans*-[PtCl$_2$(*N,N*-dimethylamine)(isopropylamine)], *cis*-DDP and *trans*-DDP.

**MATERIALS AND METHODS**

*Biological reagents and drugs.* 100-mm culture and micro well plates were obtained from NUNCLON (Roskilde, Denmark): MTT (3-[4,5-dimethylthiazol-2-yl]-2,5-diphenyltetrazolium bromide) was purchased from Sigma. FCS was supplied by GIBCO-BRL. *cis*-DDP and *trans*-DDP were purchased from Sigma. *Trans*-[PtCl$_2$(*N,N*-dimethylamine)(isopropylamine)] was synthesized as previously reported [10]. Stock solutions of the compounds (1 mg/ml) in DMEM medium (Dulbecco's modified Eagles Medium) were freshly prepared before use.

*Cell Lines and Culture Conditions.* The pair of human ovarian tumor cell lines (A2780/A2780cisR) were cultured in DMEM medium (Dulbecco's modified Eagles Medium) supplemented with 10% FCS (foetal calf serum) together with 2 mM glutamine, 100 units/ml penicillin, and 100 mg/ml streptomycin at 37°C in an atmosphere of 95% of air and 5% $CO_2$.

*Drugs Cytotoxicity.* Cell death was evaluated by using a system based on the tetrazolium compound MTT which is reduced by living cells to yield a soluble formazan product that can be assayed colorimetrically [14]. Exponentially growing A2780 and A2780cisR cells [15] were plated in 96-well sterile plates, at a density of $10^4$ cells/well in 100 µl of medium, and were incubated for 3-4 hours. Stock solutions of the compounds dissolved in DMEM were added to the wells at final concentrations from 0 to 300 µM, in a volume of 100µl/well. After twenty-four hours of incubation, 50 µl of a freshly diluted MTT solution (1/5 in culture medium) was added to a final concentration of 1 mg/ml into each well and the plate was further incubated for 5 hours. Cell survival was evaluated by measuring the absorbance at 520 nm, using a Whittaker Microplate reader 2001. $IC_{50}$ values (drug concentrations that induces 50% of cell death) were calculated from curves constructed by plotting cell survival (%) versus compound concentration (µM). All experiments were made in quadruplicate.

*Quantification of apoptosis by annexin V binding and flow cytometry.* A2780 and A2780*cisR* cells were exposed to 2x$IC_{50}$ of the platinum drugs for 24 hours. Attached and detached cells were recovered, mixed and resuspended in annexin V binding buffer (PharMingen). 2.5 µl of propidium iodide (PI, Sigma) and 1 µg/ml of annexin V-fluorescein isothiocyanate (PharMingen) were added, and the cells were left at room temperature before flow cytometric analysis in a FACScalibur Beckton-Dickinson apparatus. The percentage of apoptotic cells induced by each platinum drug (percentage of annexin V-positive/PI negative cells) was calculated from the annexinV/PI scattergrams.

*Measurements of platinum accumulation.* Cultures plates containing exponentially growing A2780cisR cells in 10 ml of DMEM medium (cell density = 2 x $10^5$ cells/ml) were exposed to 50 μM of the platinum drugs dissolved in DMEM medium for 1, 3 or 24 hours. Cells were washed with ice-cold PBS, scraped and resuspended in 700 μl of lysis buffer containing 20 mM Tris.HCl, pH 7.5, 2 mM EDTA and 0.4% Triton X-100, incubated at 4°C for 15 min and centrifuged at 12.000 rpm for 15 min in a centrifuge. Afterwards, supernatants were treated for 3 hours at 37°C with 20 μg/ml of proteinase K (Boehringer). The platinum content in the samples was determined by TXRF (total reflection X-ray fluorescence). Experiments were carried out in triplicate.

*Determination of platinum binding to DNA in vivo.* Culture plates containing exponentially growing A2780cisR cells in 10 ml of DMEM medium (cell density = 2 x $10^5$ cells/ml) were exposed to 50 μM of the platinum drugs dissolved in DMEM. The plates were incubated for 1, 3 or 24 hours under the conditions described above. Following drug incubation, culture medium was removed from the plates and the cell plates were washed with PBS. Subsequently, the cells were lysed with 700 μl of a buffer solution containing 150 mM Tris.HCl pH 8.0, 100 mM EDTA and 100 mM NaCl, incubated for 15 minutes at 4°C and centrifuged at 12.000 rpm for 15 min in a microfuge. Supernatants were treated for 3 hours at 37°C with 20 μg/ml of proteinase K (Boehringer). Afterwards, supernatants were incubated for 16 hours at 37°C with 100μg/ml of RNase A (Boehringer). Finally, DNA was extracted with a volume of phenol-chloroform-isoamyl alcohol (50 + 49 + 1), precipitated with 2.5 volumes of cold ethanol and 0.1 volumes of 3 M sodium acetate, washed with 75% of ethanol, dried and resuspended in 1 ml of water. The DNA content in each sample was measured by UV spectrophotometry at 260 nm in a Shimadzu UV-240 spectrophotometer and platinum bound to DNA was determined by TXRF. Experiments were carried out in triplicate.

*Total reflection X-ray fluorescence measurements.* The analysis by TXRF was performed using a Seifert Extra-II spectrometer (Seifert, Ahrensburg, Germany). TXRF determinations were carried out according to a procedure previously reported [16]. Briefly, a 100 μl sample of either cell supernatants or cellular DNAs from the cell cultures was introduced in a test tube of 2 ml. This solution was standardised with 100 ng/ml of Vanadium [Merck (Darmstadt,Germany) ICP Vanadium standard solution]. Afterwards, the sample was introduced into a high-purity nitrogen flow concentrator at a temperature of 70°C until the volume was reduced five times. An aliquot of 5 μl was then taken, deposited on a previously clean quartz-made reflector and dried on a ceramic plate at a temperature of 50°C. The entire process was done in a laminate flow chamber (Model A-100). The samples were analysed following the X-ray Molybdenum line under working conditions of 50 kV and 20 mA with a live-time of 1000 s and a dead time of 35%. Spectra were recorded between 0 and 20 keV. The following 15 elements were simultaneously analysed: P, S, K, Ca, V, Fe, Cu, Zn, As, Br, Rb, Sr, Ni, Mn and Pt, in order to obtain a correct deconvolution of profiles associated with the general spectrum. The Pt line was used for Pt quantification. The analytical sensitivity of the TXRF measurements was 0.3 to 22.4 ng Pt in a solution volume of 100 μl, with repeatability between 2 and 8% (n = 3).

*Intracellular GSH content.* Intracellular GSH levels were determined, in A2780 and A2780cisR cells growing as specified for the Cytotoxicity tests. Approximately 5 x$10^5$ cells/ml were seeded into P100 plates, and, after overnight incubation, cells were washed twice with ice-cold PBS. Cellular GSH was then extracted using 2 ml of ice-cold 0.6% sulfosalicilic acid followed by a 10 min incubation at 4°C. Total GSH content in the extract was then determined according to the method of Griffiths[18]. Protein quantification was carried out after solubilization in 2 ml of sodium hydroxide 1M using the Lowry assay [19]. The GSH levels were expressed as nmol/mg protein.

*Depletion of GSH levels in A2780cisR cells.* A2780cisR cells were pre-exposed for 24 hours to 50 μM of L-buthionine sulfoximine (L-BSO). This resulted in an approximately 80% reduction in GSH levels [17]. The growth inhibitory effect of the platinum compounds after 24 hours of drug exposure was then determined using the MTT method.

*Statistical Analysis.* Where appropriate, statistical significance was tested using a two-tailed Student's test.

### RESULTS AND DISCUSSION

*Cytotoxic activity.*

We have tested the cytotoxic activity of trans-[$PtCl_2$(*N,N*-dimethylamine)(isopropylamine)], *cis*- and *trans*-DDP against A2780 and A2780cisR cells after a treatment period of 24 hours. Table I shows that in A2780 cells the $IC_{50}$ value of *trans*-[$PtCl_2$(*N,N*-dimethylamine)(isopropylamine)] was similar to that of *cis*-DDP (3.7 μM and 3.6 μM, respectively). In contrast, the $IC_{50}$ value of *trans*-DDP was 30-fold higher (110 μM) than those of *trans*-[$PtCl_2$(*N,N*-dimethylamine)(isopropylamine)] and *cis*-DDP. Interestingly, *trans*-[$PtCl_2$(*N,N*-dimethylamine)(isopropylamine)] had a cytotoxic activity 2.6-times and more than 13.6-times higher than *cis*-DDP and *trans*-DDP in the cisplatin resistant tumor cell line A2780cisR ($IC_{50}$ values of 22 μM, 58 μM and > 300 μM, respectively).

Because GSH is involved in intracellular detoxification of metal drugs [20], we have also evaluated the effect of GSH on the cytotoxic activity of *trans*-[$PtCl_2$(*N,N*-dimethylamine)(isopropylamine)], *cis*-DDP and *trans*-DDP by using L-BSO (L-buthionine sulfoximine) to decrease the levels of GSH in A2780cisR cells prior to drug-treatment. Our determinations of GSH intracellular content indicated that the A2780cisR cell line possesses about 6-times higher glutathione levels than its parental A2780 cell line (GSH levels: nmol/mg protein, A2780 = 8.5 ± 0. 5; A2780cisR = 50 ± = 0.8; $p<0.01$). These data are in agreement with previous data reported in the literature and indicate that the A2780cis R cell line has high intrinsic levels of GSH [13]. Interestingly, Table I shows that while potentiation of cytotoxicity in A2780cis R cells was only slight for *cis*-DDP, it was significantly high for the two *trans* complexes *trans*-[$PtCl_2$(*N,N*-dimethylamine)(isopropylamine)] and *trans*-DDP ($p<0.01$). Altogether these results indicate that *trans*-[$PtCl_2$(*N,N*-dimethylamine)(isopropylamine)] is able to circumvent cisplatin resistance in A2780cisR cells. In addition, the cytotoxicity data obtained in the presence or absence of pre-exposure to L-BSO also suggest that as previously found for other *trans*-platinum complexes [22, 23], *trans*-[$PtCl_2$(*N,N*-dimethylamine ) (isopropylamine)] may be also more susceptible to inactivation by reaction with GSH than *cis*-DDP.

**Table I.** $IC_{50}$ mean values obtained for *trans*-[$PtCl_2$(*N,N*-dimethylamine)(isopropylamine)], *cis*-DDP and *trans*-DDP against A2780 and A2780cisR cell lines for a drug-treatment period of 24 hours. (+ L-BSO) indicates that the cells were preincubated for 24 hours with 50 μM of L-buthionine sulfoximine in order to deplete cellular glutathion levels. SD=standard deviation.

| | Ovarian carcinoma cell system A2780/A2780cisR ($IC_{50}$ ±SD) | | |
|---|---|---|---|
| | **A2780** | **A2780cisR** | **A2780cisR (+LBSO)** |
| ***trans*-[$PtCl_2$(dma)(ipa)]*** | 3.7 ± 0.1 | 22 ± 3 | 5.0 ± 0.3 |
| ***cis*-DDP** | 3.6 ± 0.4 | 58 ± 4 | 50 ± 2 |
| ***trans*-DDP** | 110 ± 8 | >300 | 144 ± 10 |

******trans*-[$PtCl_2$(dma)(ipa)]=*trans*-[$PtCl_2$(*N,N*-dimethylamine)(isopropylamine)]

*Apoptosis induction.*

After a 24 hours treatment period with equitoxic doses (2x$IC_{50}$) of *cis*-DDP, *trans*-DDP and *trans*-[$PtCl_2$(*N,N*-dimethylamine)(isopropylamine)], there was a greater cell detachment from the culture plate surface in A2780 cells compared with A2780cisR cells, as revealed by phase contrast microscopy (data not shown). Cell detachment has been previously reported as an indication of apoptosis induction [11, 12]. Both detached and attached cells were mixed and assayed by a flow cytometric annexin V binding assay [24]. Annexin V binds phosphatidyl serine residues that are asymmetrically distributed to the inner plasma membrane but move to the outer plasma membrane early in apoptosis. Figure 2 shows that treatment with *trans*-[$PtCl_2$(*N,N*-dimethylamine)(isopropylamine)] induced a higher increase in the annexin V-positive/PI negative cell population (right bottom quadrant) than treatment with *cis*-DDP or *trans*-DDP both in A2780 (Fig. 2: panels B, C and D, respectively) and A2780cis cells (Fig 2. panels F, G and H, respectively). The annexin V-positive/PI negative cell population constitutes the fraction of apoptotic cells [24].

Table II shows the percentage of apoptotic cells induced by the platinum compounds in A2780 and A2780cisR cells as calculated from the scattergrams of Figure 2. As can be observed in Table II, all the platinum drugs induced a higher percentage of apoptotic cells in the A2780 line than in the A2780cisR line. Thus, *trans*-[$PtCl_2$(*N,N*-dimethylamine)(isopropylamine)] killed most of A2780 cells through apoptosis (91.97 %). Moreover, the percentage of apoptotic A2780 cells induced by *trans*-[$PtCl_2$(*N,N*-dimethylamine)(isopropylamine)] was two-times and three-times higher than those induced by *cis*-DDP (45.20%) and *trans*-DDP (29.26%), respectively. In addition, *trans*-[$PtCl_2$(*N,N*-dimethylamine)(isopropylamine)] also induced a significant percentage of apoptotic cells in the A2780cisR line (58.38%). This percentage was 1.7-times and 5.0 times higher than those induced by *cis*-DDP (34.24 %) and *trans*-DDP (10.59 %), respectively. Altogether, the results indicate that the lower the dose of drug needed to kill the cells the higher the percentage of apoptosis induction [25]. Thus, *trans*-[$PtCl_2$(*N,N*-dimethylamine)(isopropylamine)] has a higher ability to induce apoptosis than both *cis*-DDP and *trans*-DDP in the pair of cell lines A2780/A2780cisR. Inversely, the data also suggest that the higher the dose of drug needed to kill the cells the higher the percentage of induction of necrosis [25]. Thus, it is interesting to note that *trans*-DDP induced high percentages of necrosis both in

A2780 (46.08%) and A2780cisR cells (60.62%). This finding might be related with the biological inefficacy of *trans*-DDP [4].

| | A2780 | | | A2780*cis*R | | |
|---|---|---|---|---|---|---|
| | Alive (%) | Apoptotic (%) | Necrotic (%) | Alive (%) | Apoptotic (%) | Necrotic (%) |
| **Control** | 99.78 | 0 | 0.22 | 99.65 | 0.01 | 0.34 |
| **tdmaipa** | 6.92 | 91.97 | 1.11 | 7.16 | 58.38 | 34.46 |
| ***cis*-DDP** | 17.29 | 45.20 | 37.51 | 22.85 | 34.24 | 42.91 |
| ***trans*-DDP** | 24.66 | 29.26 | 46.08 | 28.79 | 10.59 | 60.62 |

**tdmaipa** = *trans*-[$PtCl_2$(*N,N*-dimethylamine)(isopropylamine)]

*Platinum accumulation in A2780cisR cells.*

Total intracellular platinum levels found in A2780cisR cells after exposure to 50 μM of the platinum drugs for 1, 3 and 24 hours are shown in Figure 3. It may observed that cellular uptake of *cis*-DDP, *trans*-DDP and *trans*-[$PtCl_2$(*N,N*-dimethylamine)(isopropylamine)] increased as a function of time in A2780cisR cells. At short periods of drug treatment cellular levels of *trans*-[$PtCl_2$(*N,N*-dimethylamine) (isopropylamine)] were lower than those of *cis*-DDP and *trans*-DDP. Thus, the intracellular levels of *cis*-DDP, *trans*-DDP and *trans*-[$PtCl_2$(*N,N*-dimethylamine)(isopropylamine)] in the A2780cisR s were 0.13, 0.12 and 0.10 $\mu mol/2x10^6$ cells after 1 hour of incubation and progressively increased to reach respectively 0.41, 0.35 and 0,12 $\mu mol/2x10^6$ cells after 3 hours of incubation and 0.42, 0.37 and 0.30 $\mu mol/2x10^6$ cells after 24 hours of incubation. Thus, after 24 hours of incubation of A2780cisR cells with a 50 μM

concentration of *cis*-DDP, *trans*-DDP and *trans*-[$PtCl_2$(*N,N*-dimethylamine) (isopropylamine)], the percentage of intracellular platinum in A2780cisR cells relative to the platinum input was 82%, 74% and 60%, respectively. These data indicate that intracellular accumulation of *trans*-[$PtCl_2$(*N,N*-dimethylamine) (isopropylamine)] in A2780cisR cells is lower than that of both *cis*-DDP and *trans*-DDP. We think that the lower cellular uptake of *trans*-[$PtCl_2$(*N,N*-dimethylamine) (isopropylamine)] relative to *cis*-DDP and *trans*-DDP might be related to its larger molecular size.

**Figure 3.** Cellular uptake of 50 μM of *trans*-[$PtCl_2$(*N,N*-dimethylamine)(isopropylamine)] (♦), *cis*-DDP ( ) and *trans*-DDP (△) in A2780cisR cells. The results are expressed as means ± SD (n=3).

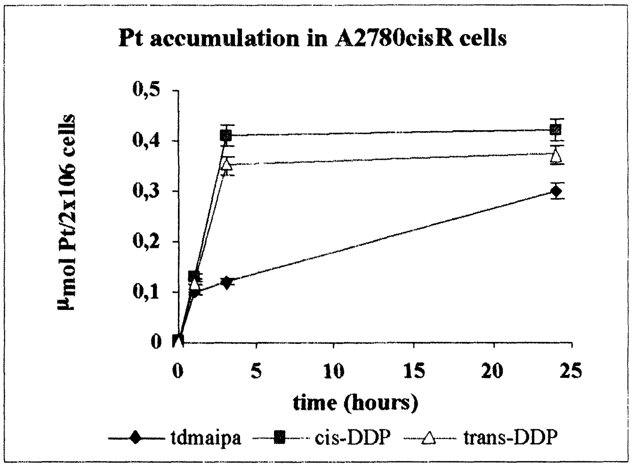

**Figure 4.** DNA binding kinetics of 50 μM of *trans*-[$PtCl_2$(*N,N*-dimethylamine)(isopropylamine)] (♦), *cis*-DDP ( ) and *trans*-DDP (△) in A2780cisR cells The results are expressed as means ± SD (n=3).

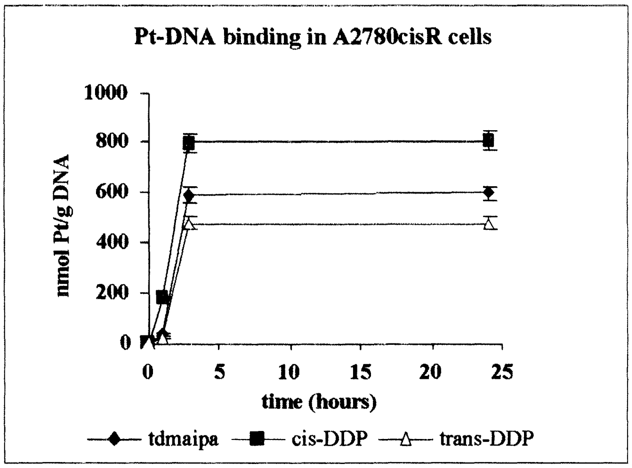

*Platinum-DNA binding in A2780cisR cells.*

Platinum-DNA binding levels in A2780cisR cells incubated with the platinum drugs (50 μM) for 1, 3 and 24 hours are shown in Figure 4. The binding of the drugs began to be quantifiable only after 1 hour of incubation. The DNA binding kinetics of *cis*-DDP, *trans*-DDP and *trans*-[$PtCl_2$(*N,N*-dimethylamine) (isopropylamine)] showed that the binding of these drugs to DNA increased as function of time in A2780cisR cells. The higher level of DNA binding was shown by *cis*-DDP. In contrast, the levels of DNA binding of *trans*-DDP were lower than those of *cis*-DDP and *trans*-[$PtCl_2$(*N,N*-dimethylamine)(isopropylamine)] at all the periods of incubation tested. The binding of *cis*-DDP, *trans*-[$PtCl_2$(*N,N*-dimethylamine)(isopropylamine)] and *trans*-DDP to DNA was respectively 180, 40 and 20 nmol/g DNA after 1 hour of incubation and progressively increased to reach respectively 810, 590 and 480 nmol/g DNA after 24 hours of incubation.

We also quantified the levels of platinum binding to DNA when the A2780cisR cells were pre-exposed to L-BSO before treatment with the platinum drugs. Figure 5 shows that under these conditions the level of DNA binding shown by *cis*-DDP, *trans*-[$PtCl_2$(*N,N*-dimethylamine)(isopropylamine)] and *trans*-DDP increased relative to that found in A2780cisR cells not pre-exposed to L-BSO. Interestingly, the higher increase in DNA binding was shown by *trans*-[$PtCl_2$(*N,N*-dimethylamine)(isopropylamine)]. Thus, the kinetics of DNA binding of *trans*-[$PtCl_2$(*N,N*-dimethylamine)(isopropylamine)] was very similar to that of *cis*-DDP in A2780cisR cells pre-exposed to L-BSO. These results support the cytotoxicity data reported above and suggests that *trans*-[$PtCl_2$(*N,N*-dimethylamine)(isopropylamine)] is significantly more affected in its reaction with DNA by cellular levels of GSH than *cis*-DDP.

**Figure 5.** DNA binding kinetics of 50 μM of *trans*-[$PtCl_2$(*N,N*-dimethylamine)(isopropylamine)] (♦), *cis*-DDP ( ) and *trans*-DDP (△) in A2780cisR cells pre-exposed for 24 hours to 50 μM of L-BSO.The results are expressed as means ± SD (n=3).

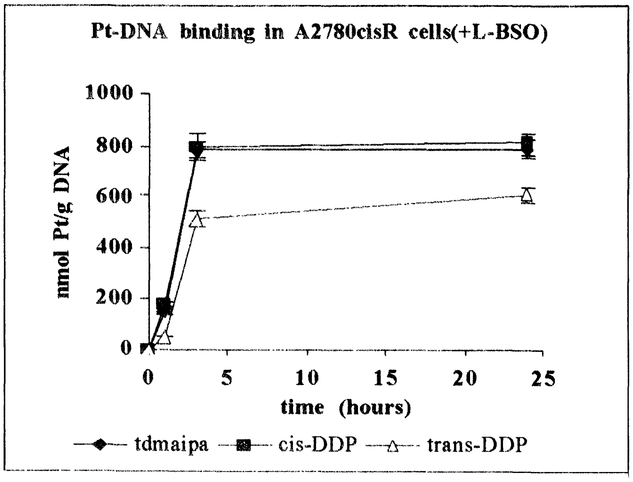

The results described in this paper show that *trans*-[$PtCl_2$(*N,N*-dimethylamine)(isopropylamine)] is able to circumvent cisplatin resistance in A2780cisR ovarian tumor cells. Thus, *trans*-[$PtCl_2$(*N,N*-dimethylamine)(isopropylamine)] exhibits greater cytotoxicity against A2780cisR cells than both *cis*-DDP (approximately 2.6-times as potent) and *trans*-DDP (more than 13.6-times as potent). Moreover, *trans*-[$PtCl_2$(*N,N*-dimethylamine)(isopropylamine)] is a better inductor of apoptosis in A2780cis R cells than both *cis*-DDP (1.7-times as better) and *trans*-DDP (5.5-times as better). These results are in agreement with previously reported data, which showed that *trans*-[$PtCl_2$(*N,N*-dimethylamine) (isopropylamine)] is also able to circumvent cisplatin resistance and induces apoptosis in Pam 212-*ras* murine keratinocytes [10, 11].

It has been reported that the A2780cisR human ovarian tumor cell line exhibits acquired resistance to *cis*-DDP from a combination of decreased uptake, enhanced DNA repair/tolerance and elevated GSH levels [12, 13]. The aim of the present study was to investigate whether the cellular uptake, the reaction with GSH and the level of platinum binding to DNA of *trans*-[$PtCl_2$(*N,N*-dimethylamine)(isopropylamine)] may help to understand the mechanism(s) by which this drug overcomes cisplatin resistance. Our results show that both the cellular uptake and the binding of *trans*-[$PtCl_2$(*N,N*-dimethylamine)(isopropylamine)] to DNA in A2780cisR cells are lower than those of *cis*-DDP. In addition, *trans*-[$PtCl_2$(*N,N*-

dimethylamine)(isopropylamine)] seems to be inactivated by reaction with GSH at a higher extent than *cis*-DDP. In fact, either the cytotoxic activity or the amount of binding to DNA of *trans*-[$PtCl_2$(*N,N*-dimethylamine)(isopropylamine)] in A2780cisR cells significantly increased when intracellular levels of GSH were depleted with L-BSO before treatment with the platinum drug. In contrast, both the cytotoxic activity and the amount of *cis*-DDP binding to DNA are not significantly altered when GSH levels in A2780cisR cells are depleted with L-BSO prior to *cis*-DDP treatment. Therefore, the intracellular accumulation, the reaction with GSH and the level of platinum binding to DNA are factors, which cannot explain the circumvention of cisplatin resistance shown by *trans*-[$PtCl_2$(*N,N*-dimethylamine) (isopropylamine)] in A2780cisR cells.

Emerging evidence suggest that an important number of cases of cisplatin resistance might be the result of the inability of *cis*-DDP to induce cell death through apoptosis in particular cell lines [8, 25]. We think that circumvention of ciplatin resistance by *trans*-[$PtCl_2$(*N,N*-dimethylamine) (isopropylamine)] might be directly related with the ability of this drug to induce apoptosis in A2780cisR cells. It is generally accepted that DNA damage and subsequent induction of apoptosis may be the primary cytotoxic mechanism of platinum drugs [26]. Although the level of binding to DNA of trans-[$PtCl_2$(*N,N*-dimethylamine)(isopropylamine)] in A2780cisR cells is lower than that of *cis*-DDP it can not be ruled out the possibility that a specific type of DNA adduct might be involved in the induction of apoptosis by *trans*-[$PtCl_2$(*N,N*-dimethylamine)(isopropylamine)]. In fact, we have previously reported that *trans*-[$PtCl_2$(*N,N*-dimethylamine)(isopropylamine)] forms a higher amount of DNA interstrand cross-links than *cis*-DDP in linear pBR322 plasmid [27]. Although the interstrand cross-links represent a minor proportion of the total lesions produced by *cis*-DDP on DNA, they have often implicated with cytotoxicity [28]. Alternatively, *trans*-[$PtCl_2$(*N,N*-dimethylamine)(isopropylamine)] might induce apoptosis in A2780cisR cells through interaction with other targets (i.e., proteins, phospholipids, cytoskeleton etc.) and subsequent cellular damage. Further research is warranted to test these two hypotheses.

### ACKNOWLEDGEMENTS

This work was supported by grants SAF00-0029 and BIO-99/1133.We also thanks the European Cost D20/0001/00 and D20/0003/00 Actions. An institutional grant from Fundación Ramón Areces is also acknowledged. We thank Johnson Matthey plc. for their generous gift of $K_2PtCl_4$.

### REFERENCES

1. Rosenberg B. *Cancer*, **55**, 2303-2316, 1985.
2. O'Dwyer P.J., Stevenson J.P. and Johnson S.W. In *Cisplatin. Chemistry and biochemistry of a leading anticancer drug,* Lippert, B., Ed.; Wiley-VCH, Basel, Switzerland, 1999; pp 31-72.
3. Kelland L.R. *Crit Rev Oncol/Hematol*, **15**, 191-219, 1993.
4. Jamieson E.R. and Lippard S.J. *Chem Rev*, **99**, 2467-2498, 1999.
5. Gately D.P. and Howell S.B. *Brit J Cancer*, **67**, 1171-1176, 1993.
6. Di Blasi P., Bernareggi A., Beggiolin G., Piazzoni L., Menta E. and Formento M.L. *Anticancer Res*, **18**, 3113-3118, 1998.
7. Andrews P.A. and Howell S.B. Cancer Cells, **2**, 35-43, 1990.
8. Pérez R.P. *Eur J Cancer*, **34** , 1535-1544, 1998.
9. Pérez J.M., Fuertes M.A., Alonso C. and Navarro-Ranninger C. *Crit Rev Oncol/Hematol*, **35**, 109-120, 2000.
10. Montero E.I., Díaz S., González-Vadillo A.M., Pérez J.M., Alonso C. and Navarro-Ranninger C. J *Med Chem*, **42**, 4264-4268, 1999.
11. Pérez, J.M., Montero, E.I., González, A.M., Alvarez-Valdés, A., Alonso, C. and Navarro-Ranninger, C. *J Inorg Biochem*, **77**, 37-42, 1999.
12. Kelland L.R., Barnard C.F.J., Mellish K.J., Jones M., Godard P.M., Valenti M., Bryant A., Murrer B.A. and Harrap K.R. *Cancer Res*, **54**, 5618-5622, 1994.
13. Kelland L.R. and Barnard C.D.J. *Drugs Fut*, **23**, 1062-1065, 1999.
14. Alley M.C. and Boyd. M.R. *Cancer Res*, **48,** 589-601, 1998.
15. Kelland L.R., Abel G., McKeage M.J., Jones M., Goddard P.M., Valenti M., Murrer B.A. and Harrap K.R. *Cancer Res*, **53**, 2581-2586, 1993.
16. Fernández-Ruíz R., Tornero J.D., González, V.M. and Alonso C. *Analyst*, **124**, 583-585, 1999.
17. Kelland L.R., Barnard C.F.J., Mellish K.J., Jones M., Goddard P.M., Valenti M., Bryant A., Murrer B.A. and Harrap K.R. *Cancer Res.*, **54**, 5618-5622, 1994.
18. Griffiths O.W. *Anal Biochem*, **106**, 207-211, 1980.
19. Lowry O.H., Rosebrough N.J., Farr A.L. and Randall R.J. *J Biol Chem*, **193**, 265-275, 1951.

20. Pendyala L., Creaven P.J., Pérez R., Zdanowicz J.R. and Raghavan D. *Cancer Chemother Pharmacol*, **36**, 271-278, 1995.

21. Mistry P., Kelland L.R., Abel G., Sidhar S. and Harrap K.R. *Brit J Cancer*, **64**, 215-220, 1991.

22. Andrews P.A., Murphy M.P. and Howell S.B. *Cancer Res*, **45**, 6250-6253, 1985.

23. Goddard P., Valenti M. and Kelland L.R. *Anticancer Res*, 14, 1065-1070, 1994.

24. Van Engeland M., Nieland L.J.W., Ramaekers F.C.S., Schutte B. and Reutenlingspergcr C.P.M. *Cytometry*, **31**, 1-9, 1998.

25. González V.M., Fuertes M.A., Alonso C. and Pérez, J.M. *Mol Pharmacol*, 2001 (in press).

26. Eastman A. In *Cisplatin. Chemistry and biochemistry of a leading anticancer drug*, Lippert, B., Ed.; Wiley-VCH, Basel, Switzerland, 1999; pp 111-134.

27. Pérez J.M., Montero E.I., González A.M., Solans X., Font-Bardía M., Fuertes M.A., Alonso C. and Navarro-Ranninger C. *J Med Chem*, **43**, 2411-2418, 2000.

28. Leng M. and Brabec V. In DNA adducts: Identification and biological significance. Hemminki K, Dipple A, Shuker DEG, Kadlubar FF, Segerbäck d and Bartsch H Eds. IARC ScientificPpublications No 125, Lyon, International Agency for Research on Cancer, 1994, pp 339-348.

